# Recognition, Signal Transduction, and Amplification of Nucleic Acids Under Isothermal Condition

**DOI:** 10.1002/ggn2.202500057

**Published:** 2026-02-22

**Authors:** Zhaohui Qin, Jiaxin Li, Xin Su, Huiyu Liu

**Affiliations:** ^1^ Beijing Advanced Innovation Center for Soft Matter Science and Engineering State Key Laboratory of Organic–Inorganic Composites Beijing Laboratory of Biomedical Materials, Bionanomaterials and Translational Engineering Laboratory Beijing Key Laboratory of Bioprocess Beijing University of Chemical Technology Beijing P. R. China

**Keywords:** amplification strategies, isothermal nucleic acid detection, recognition, signal transduction

## Abstract

Isothermal nucleic acid detection has become a compelling alternative to PCR, enabling rapid analysis with minimal instrumentation and strong potential for point‐of‐care use. Central to assay performance are three interconnected processes: recognition, signal transduction, and amplification. Recognition strategies now extend beyond base pairing to include engineered proteins, catalytic nucleic acids, and synthetic modules. Signal transduction has advanced through orthogonal chemistries and functional nanomaterials, converting molecular events into optical, electrical, or colorimetric outputs. Amplification encompasses not only nucleic acid replication but also catalytic cycles and cascade reactions that magnify signals across molecular and material scales. These innovations are converging toward integrated, programmable platforms that support multiplexing and digital readouts. In this Perspective, we highlight emerging mechanisms and design principles that define isothermal detection, and outline future opportunities to achieve accurate, scalable, and accessible diagnostics for clinical, environmental, and translational applications.

## Graphical abstract

This Perspective outlines a modular framework for isothermal nucleic acid detection, highlighting recognition and signal transduction as central determinants of assay performance, with amplification strategies serving as sensitivity enhancers. Emerging design principles and future opportunities for scalable and accessible diagnostics are discussed.

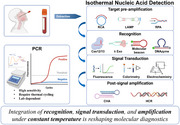

## 1 Introduction

Nucleic acid detection is central to modern life sciences and medicine, with critical roles in clinical diagnostics, infectious disease surveillance, cancer biomarker monitoring, genetic testing, and environmental safety [1]. The ability to sensitively and specifically detect nucleic acid biomarkers has not only transformed the early diagnosis and management of diseases but also enabled precise monitoring of public health and biosafety risks. As such, continuous innovation in nucleic acid detection technologies remains essential for advancing both fundamental research and translational applications [2].

**FIGURE 1:**
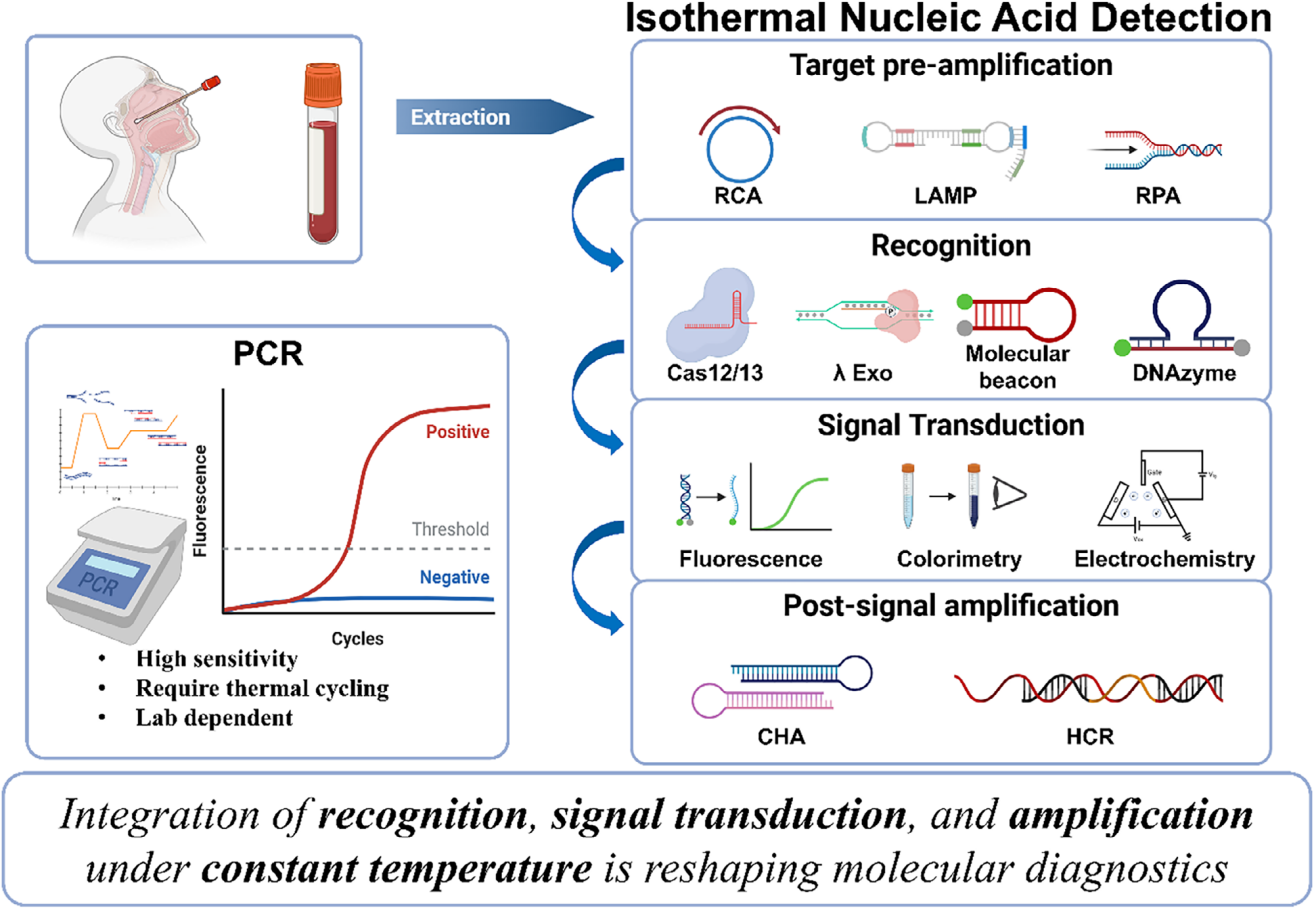
Conceptual framework of isothermal nucleic acid detection. Compared to PCR, which requires thermal cycling and complex instrumentation, isothermal strategies integrate nucleic acid recognition, signal transduction, and amplification under constant temperature. This schematic highlights the modular nature of isothermal approaches and their potential to overcome the limitations of PCR‐based diagnostics.

Despite the wide adoption of polymerase chain reaction (PCR) as the gold standard, its dependence on thermal cycling and sophisticated equipment limits deployment in decentralized or resource‐constrained settings, hindering point‐of‐care testing [3]. These challenges have catalyzed growing interest in isothermal strategies, where nucleic acid recognition, signal transduction, and amplification can be achieved under constant temperature [4]. Analogous to PCR, primer‐based isothermal amplification methods (e.g., RPA and LAMP) function as the primary engines for target enrichment, while additional recognition and signal‐processing modules are often incorporated to enhance specificity and readout [5]. In this Perspective, we review key tools for isothermal nucleic acid detection, distill emerging design principles, and outline future directions toward accessible, accurate, and scalable diagnostic platforms. We emphasize that recognition and signal transduction constitute the core determinants of assay performance, while amplification serves as an auxiliary strategy when sensitivity is limiting. Isothermal amplification approaches are discussed as two functionally distinct categories: target pre‐amplification, which increases analyte abundance prior to recognition, and post‐signal amplification, which enhances readout intensity downstream of signal generation. Figure 1 summarizes this modular architecture and the information flow across these components.

## 2 Recognition

Enzyme‐mediated strategies underpin many isothermal nucleic acid detection platforms due to the high specificity and catalytic efficiency of nucleases. Beyond primer‐based recognition inherent to target amplification, many isothermal platforms incorporate secondary recognition or verification modules to improve specificity and enable variant discrimination. Notably, CRISPR‐Cas effectors such as Cas12a [6] and Cas13a [7], as well as λ exonuclease [8], can recognize double‐stranded DNA (dsDNA), enabling direct interrogation of genomic or amplified targets. Cas12a [9] amplifies signals via collateral trans‐cleavage activity but is constrained by protospacer adjacent motif (PAM) requirements [10], whereas Cas13a [11] depends on protospacer flanking sites (PFS) [12], limiting access to targets lacking the requisite motifs. In contrast, the recently characterized activity of λ exonuclease shows no such sequence dependency, broadening its applicability across diverse dsDNA substrates and enhancing its utility for variant detection and pathogen diagnostics. Unlike its canonical activity, this newly discovered behavior enables 5′‐phosphorylated guide DNA (pDNA) to invade complementary sites within duplex DNA, triggering λ exonuclease‐mediated self‐degradation. This sequence‐independent mechanism overcomes the motif constraints of CRISPR systems and provides a versatile platform for double‐stranded DNA recognition under isothermal conditions. Looking ahead, λ exonuclease holds promise for in vitro diagnostic imaging, including spatial mapping of genomic loci and mutation analysis in fixed cells or tissues. Moreover, its programmable duplex‐targeting capability suggests potential in vivo applications in genome editing and DNA regulation. With further optimization in guide design and delivery strategies, λ exonuclease‐based systems may bridge nucleic acid sensing and genome engineering.

In parallel, nucleic acids themselves serve as programmable recognition elements through predictable base pairing and catalytic activity. Molecular beacons [13] exploit hairpin disruption upon target hybridization to generate fluorescence [14], enabling highly specific detection even at the single‐nucleotide level and making them valuable for genotyping and mutation analysis, although background fluorescence and limited multiplexing capacity can restrict their utility [15]. DNAzymes [16], identified through in vitro selection [17], combine substrate‐binding arms with catalytic cores to achieve sequence‐dependent cleavage or signal transduction, and have been employed in fluorescent or colorimetric assays as protein‐free alternatives [18]. These modules offer advantages such as facile chemical synthesis, thermal stability, and compatibility with amplification circuits, but typically exhibit lower catalytic efficiency than natural enzymes and may require specific cofactors. Together, these approaches illustrate how nucleic acids can function directly as recognition modules, expanding the design space of isothermal diagnostics beyond protein‐driven systems.

Engineered and synthetic recognition modules are rapidly emerging to overcome the intrinsic limitations of natural systems. Advances in protein engineering have yielded compact Cas variants with relaxed PAM requirements and enhanced collateral activity, enabling more flexible and multiplexed designs [11, 19]. In parallel, rationally designed DNAzymes, ribozymes, and peptide‐nucleic acid hybrids expand the programmable recognition toolbox, while synthetic biology circuits introduce logic‐based decision making [20]. Hybrid protein‐nucleic acid architectures further support modular, “plug‐and‐play” sensing strategies.

Enzyme‐driven and nucleic acid‐driven recognition strategies differ fundamentally in specificity, target type, and sequence dependence. Enzyme‐based systems, including CRISPR‐Cas nucleases and the recently discovered duplex‐targeting activity of λ exonuclease, offer high specificity and intrinsic catalytic signal amplification, enabling direct recognition of double‐stranded DNA. However, CRISPR effectors remain constrained by PAM or PFS requirements, whereas λ exonuclease exhibits minimal sequence dependence, expanding target accessibility for genomic analysis. In contrast, nucleic acid‐driven modules such as molecular beacons and DNAzymes rely on base pairing for recognition, providing excellent single‐base resolution but are largely restricted to single‐stranded or structurally accessible regions. Together, enzyme‐driven approaches favor robust duplex targeting with catalytic gain, while nucleic acid‐based strategies prioritize programmability and fine discrimination, underscoring their complementary roles in isothermal diagnostics.

Given that the majority of genetic information resides in double‐stranded DNA, recognition strategies capable of directly targeting duplex substrates are particularly attractive for diagnostic applications. Such approaches bypass denaturation or extensive preprocessing, simplifying workflows and preserving the native genomic context. Enzyme‐based systems, including CRISPR‐Cas nucleases and λ exonuclease, exemplify this capability, enabling pathogen detection and variant analysis directly from dsDNA. Compared with single‐stranded recognition, dsDNA targeting aligns more closely with clinical samples, but faces obstacles such as PAM or PFS constraints, background from nonspecific cleavage, and difficulties in resolving single‐nucleotide variants within stable duplexes. Thus, while offering a more direct route to genomic analysis, dsDNA recognition requires careful design to balance accessibility, specificity, and signal robustness.

## 3 Signal Transduction

Signal transduction focuses on converting molecular events into measurable outputs, whereas post‐signal amplification refers to mechanisms that enhance signal intensity prior to or during readout. Following molecular recognition, the next essential step in isothermal nucleic acid detection is the conversion of these recognition events into measurable signals. Signal transduction serves as the bridge that links molecular binding to readable outputs, thereby defining the sensitivity, robustness, and usability of diagnostic platforms.

Optical signal transduction provides versatile strategies to convert nucleic acid recognition events into measurable outputs, leveraging mechanisms such as fluorescence, colorimetry, and luminescence. Fluorescence readouts are commonly achieved through signal quenching or Förster resonance energy transfer (FRET), where structural rearrangements or hybridization‐induced conformational changes modulate emission intensity. These approaches offer high sensitivity, real‐time monitoring capability, and the potential for multiplexed detection through multi‐color channels, but they often require sophisticated optical equipment and are susceptible to background interference from autofluorescence and light scattering. Colorimetric strategies provide an alternative [21], enabling direct visual readout by the naked eye. Enzyme‐mimicking nanomaterials (nanozymes) or G‐quadruplex/hemin complexes catalyze chromogenic substrate reactions to generate distinct color changes [22], while gold nanoparticle (AuNP) aggregation induces plasmonic shifts that produce easily distinguishable signals [23]. These assays are low‐cost, rapid, and suitable for resource‐limited or field settings, though they generally suffer from lower sensitivity and limited quantitative precision. Beyond fluorescence and colorimetry, chemiluminescence and bioluminescence offer self‐emitting readouts with inherently high signal‐to‐noise ratios and scalable reporting formats. However, their reliance on labile substrates and enzymatic stability may constrain widespread deployment. Collectively, optical signal transduction modalities provide a diverse toolbox, each with context‐dependent trade‐offs between sensitivity, portability, cost, and multiplexing capability.

Electrical signal transduction translates nucleic acid recognition into measurable current, voltage, or impedance changes, typically through electrode‐based interfaces [24]. Hybridization events or enzymatic reactions can alter surface charge, conductivity, or interfacial capacitance, producing highly sensitive readouts that are readily compatible with miniaturized sensors [25]. These methods enable label‐free detection, low sample consumption, and straightforward integration with portable electronic devices, making them attractive for point‐of‐care testing [3]. Moreover, the use of nanomaterials such as graphene, carbon nanotubes, or metallic nanoparticles can significantly enhance post‐signal amplification and lower detection limits. Nonetheless, challenges remain: electrode surface fouling and nonspecific adsorption often compromise reproducibility in complex biological samples; instrumentation may require careful calibration; and multiplexing is less straightforward compared to optical channels. Despite these constraints, electrical transduction offers a robust and scalable pathway toward miniaturized, cost‐effective, and field‐deployable nucleic acid diagnostics.

Beyond molecular‐level amplification, cross‐scale strategies further enhance detection sensitivity and precision. Single‐molecule detection [26] allows direct monitoring of individual recognition events [27], achieving unmatched sensitivity though often requiring advanced optics or microfluidics [28]. Digital assays, such as digital RPA [29] or digital CRISPR [30, 31] platforms, partition reactions into nanoliter or picoliter compartments, providing absolute quantification from binary readouts and enabling applications in rare mutation analysis and viral load monitoring. Beyond compartmentalization, emerging CRISPR‐based diagnostic systems such as SHINE‐TB integrate isothermal amplification with Cas‐mediated detection in simplified, often one‐pot formats, enabling rapid, field‐deployable tuberculosis diagnosis. These platforms emphasize minimal instrumentation, lyophilized reagents, and visual or lateral‐flow readouts, illustrating a parallel trend toward highly integrated, user‐friendly CRISPR diagnostics that complement digital strategies by prioritizing accessibility and clinical translation. Array‐based formats, including microarrays and microchamber systems, expand throughput by enabling parallel detection of multiple targets, which is particularly valuable for pathogen surveillance, environmental monitoring, and multiplexed clinical diagnostics. These cross‐scale approaches, while frequently device‐dependent, establish critical links between molecular recognition and high‐throughput readouts, advancing scalable and quantitative diagnostic technologies.

Overall, signal transduction strategies offer diverse routes for converting molecular recognition into accessible outputs. Optical methods excel in intuitive readouts and point‐of‐care use, while electrical platforms provide strong potential for integration with digital devices. Future opportunities lie in improving robustness in complex biological environments and exploring hybrid modalities that combine complementary strengths, paving the way for versatile and widely deployable isothermal nucleic acid diagnostics.

## 4 Amplification

In isothermal detection, amplification may encompass target pre‐amplification and post‐signal amplification. Primary target amplification of nucleic acids functions primarily as a pre‐amplification strategy to boost target abundance before signal readout. For DNA targets, classical enzymatic approaches include rolling circle amplification (RCA) [32], recombinase polymerase amplification (RPA) [33], and loop‐mediated isothermal amplification (LAMP) [34]. RCA generates long single‐stranded products from a circular template [35], providing a strong signal output but requiring prior template preparation [36]. RPA operates at near‐physiological temperatures (37–42°C) with rapid turnaround [37], well suited for point‐of‐care applications [38]. LAMP employs multiple primers for exponential amplification under isothermal conditions [39], offering high sensitivity within an hour, though with increased primer design complexity [40]. For RNA targets, nucleic acid sequence‐based amplification (NASBA) [41] and transcription‐mediated amplification (TMA) [42] integrate reverse transcription with RNA polymerase‐driven amplification to generate RNA copies at 37–41°C [43], combining specificity with compatibility for downstream detection [44]. Collectively, these methods provide flexible options depending on the balance of speed, sensitivity, and operational simplicity.

Although highly sensitive, pre‐amplification are intrinsically vulnerable to non‐specific amplification, leading to compromised robustness and false‐positive signals. Some of the strategies are particularly susceptible to primer‐dimer formation, off‐target hybridization, and background leakage under low‐stringency conditions. Current mitigation strategies, including rational primer design, enzyme engineering for enhanced mismatch discrimination, and digital partitioning, effectively suppress background amplification and enable absolute quantification, thereby significantly improving assay reliability and clinical translatability.

Post‐signal amplification transforms limited molecular recognition events into measurable outputs with enhanced sensitivity. Enzyme‐free approaches, such as hybridization chain reaction (HCR) [45] and catalytic hairpin assembly (CHA) [46], rely on programmable hybridization cascades to generate extended DNA structures or catalytic turnover without enzymatic input [47, 48]. These strategies offer robustness and modularity but often suffer from slower kinetics and reduced efficiency in complex matrices [49, 50, 51]. Enzyme‐driven amplification, by contrast, encompasses cyclic turnover reactions, exponential amplification reaction (EXPAR) [52, 53, 54], and cascade enzymatic processes, which achieve rapid and efficient signal gain but depend on enzyme stability and supply [55]. Nanomaterial‐assisted amplification, leveraging catalytic entities such as nanozymes or G‐quadruplex/hemin complexes, further broadens the repertoire by coupling catalytic activity with optical or electrochemical outputs [56]. Together, enzyme‐free approaches prioritize programmability and stability, while enzyme‐driven systems excel in speed and sensitivity, enabling adaptable amplification schemes for diverse diagnostic contexts.

## 5 Integration and Applications

Point‐of‐care testing(POCT) represents a primary application domain for isothermal nucleic acid diagnostics, driven by the demand for rapid, low‐cost, and user‐friendly platforms deployable outside of centralized laboratories [57]. By leveraging portable devices, lateral flow strips, and smartphone‐based readouts, these systems allow real‐time analysis at the bedside, in field settings, or in low‐resource environments. The isothermal nature of the assays eliminates the need for bulky thermocyclers, enabling compact hardware integration and even electricity‐free operation through body heat or chemical heating packs. Such portability is critical for decentralized healthcare delivery, particularly in rural clinics, outbreak response, and at‐home testing. Challenges remain in balancing assay robustness with device simplicity, including stable reagent storage under ambient conditions and reliable detection across diverse sample types. Continued innovation in lyophilized reagents, microfluidic integration, and digital connectivity is expected to further advance POCT platforms, ensuring broader accessibility and clinical impact [3].

Simultaneous detection of multiple nucleic acid targets is critical for differential diagnosis, pathogen surveillance, and comprehensive genotyping. Orthogonal systems based on CRISPR‐Cas effectors with distinct recognition requirements or DNAzyme circuits with sequence‐specific catalytic cores provide the molecular basis for multiplexing [58, 59, 60]. Optical strategies, including the use of spectrally distinct fluorophores or colorimetric barcodes, enable parallel signal readouts, while spatially resolved platforms such as microarrays or droplet‐based digital assays expand throughput to hundreds or thousands of targets [61]. Multiplexed designs must address crosstalk, reagent compatibility, and quantitative accuracy, particularly when analyzing complex clinical or environmental samples [62]. Recent advances in programmable recognition and modular signal transduction are driving scalable multiplex assays, offering high information content for multi‐pathogen detection, antimicrobial resistance profiling, and precision medicine applications [63].

Isothermal nucleic acid diagnostics have been widely applied in both clinical and environmental contexts, leveraging rapid turnaround and high sensitivity. In clinical settings, these assays enable early detection of infectious agents, monitoring of viral load, liquid biopsy for cancer biomarkers, and surveillance of antimicrobial resistance genes [64]. Environmental applications include water and food safety testing, monitoring of pathogenic microorganisms, and detection of genetic pollutants or invasive species [55]. Compatibility with minimal sample preparation and diverse sample types, such as blood, saliva, wastewater, or environmental swabs, facilitates deployment in routine monitoring and outbreak response. Although performance can be influenced by sample complexity and potential inhibitors, ongoing improvements in pre‐treatment methods, signal amplification, and portable readout systems continue to expand applicability and support timely decision‐making in healthcare and ecological surveillance.

Integration of isothermal nucleic acid assays with microfluidic devices and automated sample processing enables fully enclosed, sample‐to‐answer systems that minimize human intervention and reduce contamination risk [65]. Automation improves reproducibility and allows precise control of reaction conditions, facilitating consistent performance across multiple samples. Coupling these platforms with artificial intelligence algorithms enhances data interpretation by analyzing fluorescence or colorimetric readouts, identifying subtle trends, and enabling real‐time decision support [66]. Such smart diagnostic systems are particularly valuable for high‐throughput screening, epidemic monitoring, and personalized medicine, where rapid and accurate data interpretation is essential [67]. The combination of automation and computational analysis not only increases throughput and reliability but also broadens accessibility, enabling deployment in laboratories, clinics, and remote locations without specialized personnel [68].

Extending isothermal nucleic acid detection from in vitro assays to intracellular and in vivo applications is a key frontier for next‐generation biosensing. Programmable recognition modules such as CRISPR effectors [69, 70, 71] and DNAzymes [72], and other synthetic nucleic acid sensors [73] offer compact and sequence‐specific sensing, yet their translation is limited by delivery barriers, nuclease degradation, and reduced activity under physiological conditions. Recent discovery of ultra‐compact CRISPR systems, including CasΦ, Cas12f, and Cas14, provides new opportunities for intracellular sensing due to their small size, reduced delivery burden, and, in some cases, relaxed target constraints. These features make them particularly attractive for viral delivery, intracellular imaging, and genome interrogation. Fluorescence and luminescence remain the most practical live‐cell readouts, but are limited by tissue penetration, autofluorescence, and signal attenuation. Overcoming these challenges will require integrated advances in probe stabilization, delivery strategies, and background suppression to bridge the gap between in vitro performance and in vivo applicability.

Computational and AI‐assisted tools are increasingly reshaping isothermal nucleic acid diagnostics by transforming raw signals into reliable decisions and guiding system design. Machine learning enables early classification of amplification kinetics and discrimination between true and nonspecific signals, while smartphone‐based image analysis standardizes colorimetric readouts in point‐of‐care settings. Beyond data interpretation, AI‐driven models are being applied to enzyme engineering, probe sequence optimization, and rational integration of recognition, amplification, and readout modules, accelerating performance tuning and system‐level design. Deep‐learning frameworks further support signal denoising and multiplex deconvolution in low signal‐to‐background environments. Collectively, these approaches enhance analytical robustness, scalability, and automation, expediting the translation of isothermal assays into practical diagnostic platforms.

## 6 Concluding Remarks

Despite significant advances, key barriers still hinder the clinical translation of isothermal nucleic acid diagnostics, including nonspecific amplification, instability of recognition elements in complex matrices, fluctuating enzyme performance, and the potential cytotoxicity of certain reporters and nanomaterials. Reliable single‐nucleotide discrimination and quantitative accuracy across diverse sample types, therefore, remain major bottlenecks.

Looking ahead, enzyme‐based modules that integrate programmable recognition with catalytic amplification, particularly CRISPR systems and emerging exonuclease‐enabled strategies, are likely to remain central to platform development. In parallel, compact and protein‐free systems such as DNAzymes and synthetic nucleic acid circuits may gain importance in applications emphasizing stability, simplicity, or intracellular compatibility. Future progress will likely rely on modular architectures that combine complementary modules rather than a single universal solution.

Beyond amplification‐dependent schemes, amplification‐free strategies using plasmonic nanomaterials, transistor‐based sensors, and single‐molecule imaging are emerging as promising alternatives to reduce false positives in complex samples. Achieving truly amplification‐free detection will require high‐affinity probes coupled with ultrasensitive hardware, where direct dsDNA recognition combined with single‐molecule or electronic readouts may enable simplified yet highly accurate genomic analysis.

At the systems level, spatial partitioning, spectral barcoding, and orthogonal signal outputs will drive high‐throughput multiplexing for surveillance and precision diagnostics. Integration with microfluidics, automation, and machine‐learning‐based interpretation will further improve robustness and usability. While isothermal methods are unlikely to replace PCR as a universal laboratory standard, they are well‐positioned to complement and, in decentralized and point‐of‐care settings, potentially outperform PCR‐based diagnostics.

## Conflicts of Interest

The authors declare no conflicts of interest.
